# Supplementary material for: Causal Relationship between Aging and Anorexia Nervosa: A White-Matter-Microstructure-Mediated Mendelian Randomization Analysis
Source: Biomedicines. 2024 Aug 16;12(8):1874. doi: 10.3390/biomedicines12081874 (PMC11351342; doi:10.3390/biomedicines12081874)
Supplement: Supplementary file 1 [file biomedicines-12-01874-s001.zip › Supplementary Figure/Figure S8/Leaveoneout plot of MR analysis for TL to ubm-b-1981.pdf]

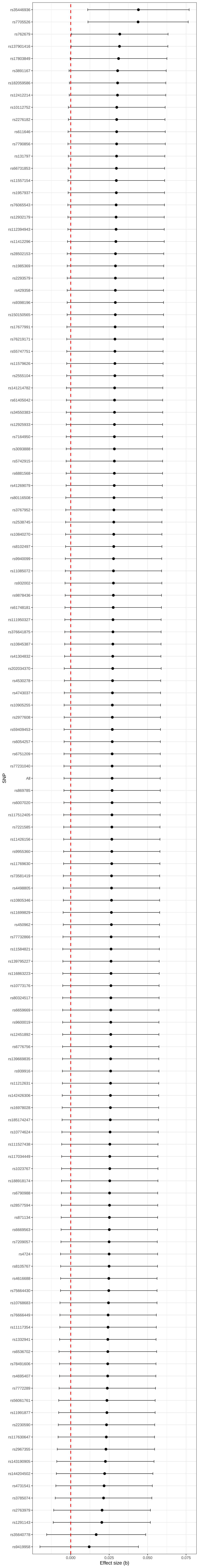

rs35446936
rs7705526
rs762679
rs137901416
rs17803849
rs3891167
rs182059586
rs12412214
rs10112752
rs2276182
rs611646
rs7790856
rs131797
rs66731853
rs11557154
rs1957937
rs76065543
rs12932179
rs112394943
rs11412296
rs28502153
rs1985369
rs2293579
rs429358
rs9398196
rs150150565
rs17677991
rs76219171
rs55747751
rs11579626
rs2555104
rs141214782
rs61405042
rs34550383
rs12925933
rs7164950
rs3093888
rs5742915
rs6881568
rs41269079
rs80116508
rs3767952
rs2538745
rs10840270
rs8102497
rs9940099
rs11085072
rs932002
rs9878436
rs61748181
rs111950327
rs376641875
rs10845387
rs41304832
rs202034370
rs4530278
rs4743037
rs10905255
rs2977608
rs59409453
rs6054257
rs6751209
rs77231040
All
rs869785
rs6007020
rs117512405
rs7221585
rs11426156
rs9955360
rs11769630
rs73581419
rs4498805
rs10805346
rs11699829
rs450962
rs77732866
rs11584821
rs139795227
rs116863223
rs10773176
rs80324517
rs6659669
rs9600019
rs12451892
rs6776756
rs139669835
rs939916
rs11212631
rs142426306
rs16978028
rs185174247
rs10774624
rs111527438
rs117034449
rs1023767
rs188918174
rs6790988
rs28577594
rs871134
rs6669563
rs7209057
rs4724
rs8105787
rs4616688
rs75664430
rs10768683
rs76666449
rs11117354
rs1332941
rs6536702
rs78491606
rs4695407
rs7772289
rs56061761
rs11991877
rs2230590
rs117630647
rs2967355
rs143190905
rs144204502
rs4731541
rs3785074
rs2763979
rs1291143
rs35640778
rs9419958
SNP
0.000
0.025
0.050
0.075
Effect size (b)
